# On the Medicinal Virtues of Marine Plants

**Published:** 1816-09

**Authors:** John Chas. Collins

**Affiliations:** One of the Physicians to the Swansea Dispensary.


*For the London Medical and Physical Journal.*

### *On the Medicinal Virtues of Marine Plants;* by John Chas. Collins, M.D. F.L.S. one of the Physicians to the Swansea Dispensary.

"Fact and repeated experiments have alone informed us, that jalap will purge, and ipecacuanha vomit; that the poppy occasions sleep; that bark will cure an ague; and that quicksilver will salivate. If we examine the whole Materia Medica, and the whole practice of physic, we shall not find one efficacious simple, nor one established method of cure, which were discovered or ascertained by any other means."—Heberden.

It is to be lamented, that, in an age remarkable for its attention and progress in the study of nature, so little has been done to render the abundant stores of marine creation applicable to disease; I am induced, therefore, by the hope of exciting the medical profession to further researches in this this interesting enquiry, to lay before them a short account of some of the more common productions, and to state the result of their effects.

The Fuci genera, which in the economy of nature seems particularly useful in affording food, shelter, and protection, to the fish and insect tribes, has also, by the ingenuity of man, been made subservient to other purposes; but as a remedy in disease they have been too much neglected; yet, from the nature of their composition, and what little we already know of their effects, it seems highly probable that discoveries of considerable importance may reward the accurate experimentalist.

The primitive food of man must have been plants, presented to him by the hand of Nature, and his first knowledge of dietetics could only extend to articles of vegetable origin; but, in his numerous experiments to produce variety, the medicinal effect of some could not fail of being brought to light, and it is but reasonable to suppose that the marine vegetable productions partook of these enquiries, and were early placed in this catalogue of remedies. Thus it has been observed "that the ancients had almost all their remedies for diseases of the glands from the sea." That marine vegetation is capable of supporting the life of land-animals, we have undisputed authority; as in Scotland and its islands, seaweeds serve as winter food for the cattle; and Linnæus informs us, that the Swedish name for the Fucus vesiculosus is *Swientang*, from its application to the fattening of swine. Many of the Fuci genera in various parts of the world are mixed with, and used as, food by man, and the Fucus edulis and palmatus are in much esteem amongst the Scotch and Irish, the Ulva umbilicalis is frequently to be seen on our own tables, and the delicious and high-priced nest of the hirundo edulis, so eagerly sought after by the eastern epicure, is said by some authors to be principally composed of marine plants.

The late Dr. James Russel added an appendix, on the use and preparation of the Quercus Marina, to his Essay on Salt Water in Glandular Complaints; but he has not been sufficiently accurate either in his description of the plant, or method of preparation; still it is the source of almost every thing which has been said on the subject by subsequent authors; and we have to lament, he never executed his promise of treating more fully on the virtues of other marine plants and submarine medicines.

It has been my wish to be particular in the direction for preparing the substances, from a conviction that the disreputable terms in which we find their virtues mentioned by

 late late writers on Scrofula is in a great measure to be attributed to want of uniformity in their preparations; for, as marine plants have roots, stalks, leaves, and seeds, it is necessary we should be as particular with them as when we use atmospheric plants.

I shall, for the present, principally confine myself to a description of the Fucus vesiculosus and nodosus, their preparation and uses; yet other marine productions are well worth investigation, as the Sponges Flustras and Sertularias; and, I have every reason to believe, that the virtues of the sponges on our own shores are equal, if not superior, to those imported from abroad. I hope, at a future period, to communicate observations on a variety of these, well entitled to the attention and trial of medical men.

It may have been expected, that, as the substances I propose to treat of here most frequently have been employed in scrofula in its various forms, I should give some account of this increasing and destructive disease, but the numerous treatises already before the public render it quite unnecessary. It cannot however be too strongly impressed on the minds of parents and the public, that, against a disease of that insidious nature, our remedies should be employed and persevered in from its very first appearance, and that the prevention should be particularly an object of our constant attention. From the extensive ravages it is daily making, we must join in the remark of a late celebrated physician, whose attention it had much engaged, that "it is lamentable to see in a nation whose every attention has been successfully employed in the improvement and symmetry of the brute creation, so little has been bestowed in promoting a similar effect by preventing and securing from disease their own *tender offspring*."

### Fucus Vesiculosus.

*Fucus Marinus*, sea-wrach, or sea-weed.

*Frond* coriacious, flat, mid-ribbed, linear, dichotomous, quite entire; vesicles spherical, crenate in the membrane of the frond; receptacles solitary, terminal, compressed, turgid, mostly elliptical; root an expanded woody callous disk.

Frond, flat winged, from one to four feet long, and from half an inch to one inch and a half wide, every-where linear, forked near the root, and afterwards repeatedly dichotomous, the intervals between each division uncertain, but mostly rather long in the lower ones, and very short in the upper; the angles of the upper dichotomus are remarkably acute, where a vesicle is placed near the axilla, in which case the ligaments are potent or even divaricated (*fucus divaric.* of Linn.); all the branches are of nearly equal height, the apices apices are rounded and not unfrequently notched, the margins quite entire; throughout the whole length of the frond runs a midrib of a blackish colour, and as thick as a goose-quill at the base, but gradually growing more pale and thin, yet so as to remain thicker and darker than the rest of the frond; the surface, especially in young plants, is perforated with minute scattered pores, from which issue small tufts of small white pointed fibres in the membranous part of the frond; throughout its whole length are found immersed spherical vesicles, varying in size from that of a pea to that of an hazel-nut, much more numerous in some individuals than in others, mostly opposite along the side of the midrib, and solitary at the base of the dichotomus, constantly placed close to the midrib, in some instances occupying the whole of the membrane, in others leaving a part flat; so they appear bordered by a small margin, externally smooth, internally empty, excepting a net-work of pellucid pointed anastomosing fibres; besides these, particularly in spring, almost always near the apices, but never, I believe, reaching completely to them, are often observable elliptical swellings (*Fucus inflat*, Linn.) an inch or two long, generally confined to the membrane on each side of the midrib, but sometimes taking in the midrib itself, so that the whole branch becomes inflated and nearly cylindrical; their colour is a pale yellowish green, their substance necessarily thin, for they originate from air generated between the double membrane of which the former consist, separating the two coats from each other; towards the root the leafy part of the frond is most frequently worn away by the action of the waves, and the naked midrib looks like a stipes; the same is also sometimes, but very rarely, the case throughout the whole length of the frond.

Fructification, compressed; turgid receptacles placed at the end of the branches, solitary, or in pairs, varying in form through all the gradations from roundish to linear, or occasionally obcordate, by no means constant even in the same specimen, but mostly elliptical, from a quarter of an inch to two inches long, generally wider than the branches, externally uneven, and perforated with very many minute pores; under which lie imbedded spherical tubercles, composed of short pointed fibres mixed with seeds of an elliptic form, surrounded with a pellucid limbus, and appearing under a powerful microscope to contain six or seven roundish grains; the center of the receptacle is filled with a colourless, tasteless, pellucid mucus, through which passes a net-work of anastomosing fibres; colour, dark olive green, paler towards the apices, smooth and glossy when dry, black and green substance, coreaceous, flexible, and tough, but brittle after it is dried.

This is one of the most common marine plants native of our shores, and varies materially in its appearance in different situations. The fresh pods begin to make their appearance about April and May, by a change of colour at the extremity of the frond; and, excepting in particular situations, the fruit has not become quite ripe until about July, when the pod breaks, and discharges its gelatinous contents, and the plant becomes withered and yellow.*

### *Preparations of the Fucus Vesiculosus.*

*Pulvis Fuci Vesiculosi.*—The method of preparing this is by selecting the fresh growing plant in the spring before there is any, or very little, appearance of fructification, washing it in salt-water so as to free it from all sand and other impurities, picking out and rejecting those parts which are covered by a species of Sertularia, hanging it up to dry in the sun, or an oven, so as gradually to evaporate the moisture it contains until it becomes dry, brittle, and capable of being powdered; care should be taken during exsiccation not to shake or handle the plant much, as it becomes covered by a copious crystallization of salts, which easily falls off. I cannot recommend this preparation, as I found it unpleasant to the taste, and not so active as the calcined plant, hereafter to be noticed. Dr. Russel says, he has given it inwardly in powder to the quantity of one drachm at a dose, and adds, that a larger was nauseous to the stomach. In one case of tænia it seemed to promote the discharge of the worm, but which was completed by the Ol. Terebinthinæ; as a common vermifuge, joined with rhubarb, and in small repeated doses, it will sometimes answer.

*Fructus Fuci Vesiculosi.*—When the mucus or juice contained in the receptaculum of this plant is required for internal use, it should be gathered quite ripe in July or August; the ends of the plant upon which the receptaculum grows, are to be cut off separately, washed in salt-water, then bruised in a marble mortar, and the juice expressed through a coarse muslin or linen bag: about one ounce will be a proper dose for an adult; when taken early in a morning in bed, it produces a gentle diaphoreses, and soon affects the bowels. This is a convenient substitute for sea-

* Vide Turner on the Fuci, vol. ii. 44, 117,—Nodosus, ii. 252, 112.
Flora Scotica, vol. ii. 904,———, ii. 918.
Holly i. 1.

water water with people who cannot be prevailed upon to take a sufficient dose of the latter, and particularly useful for children, as it is easily given; in most constitutions, it does not irritate as much as salt-water, more especially in those of a spare habit. It should be prepared fresh every morning, as it undergoes a quick change. Russel used it instead of snails with cream, drinking after it either eryngated or asses' milk; it has also this advantage over salt-water, that it does not leave the bowels in the torpid state the latter often does. In cases of habitual costiveness, and wherever a laxative is indicated, this will be found an useful substitute for those generally in use, particularly when other preparations of the plant are employed externally. It has with some people the character of being a solvent in calculous complaints; I have no doubt of it promoting the urinary secretions. From the external application, I have experienced great satisfaction in removing indolent tumours, so situated as to render it inconvenient or impossible to use a cataplasm; in long continued swellings about the joints, without inflammation, stiffness, and rigidity, or perceptible enlargement; in incipient swellings of the glands of the neck, or after they have inflamed and suppurated; for, when once a fluctuation is felt, it does not seem to be of use, except they become very indolent and of long duration. It should be applied night and morning, and the part not washed after the application, though to promote cleanliness it may be so before; the juice must be rubbed in to dryness, notwithstanding a slight eruption may have been excited. The action of friction in the use of the Fucus has been by many late authors considered as the sole cause of benefit. That it is a powerful coadjutor I will not deny; but I am satisfied, that friction is rendered, in certain cases, a much more efficient remedy by the addition of this application. The great success which attends a regular and judicious course of friction, as pursued by an ingenious surgeon at Oxford, has, in many cases, been truly astonishing; yet there are peculiar cases and constitutions in which properly selected medicines are of undoubted advantage, independent of the probability of calling forth the powers of Nature, by affecting the mind with confidence, so strongly instanced in many diseases of the human body. Children of strumous parents are frequently affected by eruptions in the bend of the joints, particularly those of the arms and knees, itching and very troublesome; in such cases, I am fully justified in strongly recommending this application. In indolent inflammation of the eye-lids, its use has been very great; in three cases of confirmed Tenea Capitis, after the strongest mercurial and other corrosive preparations

 had had been applied without completing the cure, it has been found of the greatest advantage. In two cases of incipient Bronchocele, I think it materially assisted the action of the medicine directed (Spongia Usta); and, when a cataplasm of the plant is to be applied, it should be well rubbed in, also before a common poultice.

*Linimentum Fuci Vesiculosi.*—Dr. Russel has given us his method of making this, by allowing the seed-pods to ferment in salt water, which, after keeping for some time, becomes thinner, and so sharp that it penetrates the skin immediately on application, and excites a sense of prickling. Of the vesicles of sea wrach, full of their liquor, gathered in July or August, two pounds—sea water, two pounds—let them stand together in a glass vessel ten or twelve days, until the liquor approaches the consistency of honey; then strain, and there-with rub the affected glands, chiefly when the tumour is abating, twice or thrice every day, washing the part with salt water. In those cases in which I have used this liniment, I found it produce a considerable eruption on the part to which it was applied, and, the older the preparation, the stronger this effect. I have not observed that it has a more efficacious effect than the fresh juice, but it has this advantage, that it is at all times and seasons ready, and may be conveyed to any distance required. During the winter and spring, a poultice made with this liniment and oatmeal, will prove a very useful application to indolent tumours. In some cases of scorbutic eruption of the face, incident to hard living, the application of this liniment every morning before breakfast, and washing it off an hour afterwards, effects a cure; and, in one case, for three successive years, this effect was produced by it.

*Cataplasma fuci vesiculosi.*—To form this, any quantity of the young receptacles should be gathered from the fresh plant, and freed from impurities by washing in salt water, steamed with a small quantity of sea water in a close vessel, until they soften; afterwards beat into an uniform mass to the proper consistence. It should be prepared from the plants before the receptacles come to maturity; the extremities of the plant, as early as April or May, begin to show the appearance of fruit, by a change of colour, becoming yellow, and swelled, at first being hollow, or having only those hairy fibres within, which ultimately secrete the juice, and fill it with mucus; these yellow terminations, when separated from the coriaceous part of the plant, and boiled, produce a soft saponaceous mass, the coats of the receptacles at this early age softening by heat; whereas, when made of the ripe fruit, it becomes hard. This is the preparation of the the plant in most common use, but sufficient pains are not taken to make it carefully, for, when made of the frond and whole plants, it must be, and is, much less efficacious than that which is made exclusively of the softer parts. It will keep good, when properly made, for many months, placed in a jar, and tied over with bladder.

In cases of incipient scrofulous tumours, this is an application of very considerable efficacy, and should be used every night warm, taking in the morning, as a laxative, either salt water, or one of the preparation of this plant I have here recommended for that purpose. When matter has been already formed, and not discharged, it is of less use; therefore it is either in the earlier stages, or after the ulcer has been completely established, and the discharge has taken place, that it should be applied. The changes produced in the former cases will be a gradual diminution of the swelling, attended sometimes by an eruption around it: in the latter the edges put on the appearance of healing, the matter discharged becomes more purulent, granulations arise, and a healthy cicatrix compleats the cure. It is, however, highly prudent to continue this application for some length of time afterwards, or the Fructus fuci every night. When all the usual remedies have failed, this application of the plant has been found to succeed; and, of numerous cases which have fallen under my observation, I think it quite unnecessary to relate more than the few following.

A young gentleman, residing in London, who lost his mother at an early period, and whose constitution appeared to be compleatly of the strumous temperament,—the glands in his neck enlarged, and several ulcers open,—had, for many succeeding seasons, tried the effect of a residence on the sea-side, bathing, &c. but with only temporary advantage. He was completely recovered, in London, by a regular application of this plant; and he has been quite free from any return for eight years, and, I think it probable, will escape for life.

In ulcers in the groin, occasioned by other diseases, but from the scrofulous tendency in the constitution, continuing open long after the original disease had been cured, I speak with the greatest confidence as to its efficacy, as, out of a great many similar cases, two I shall mention, which had resisted all the various means suggested by the most eminent professional men in London and Bath, and who had frequently tried a residence near, and bathing in, the sea; when an application of three weeks completed their cure.

In cases where the parts are in an high state of inflammation, or where the disease has affected the bones, or an evident evident fluctuation is present, I think the cataplasm should not be applied, as it has, in some instances, aggravated the symptoms. In enlargement of the joints, with inflammation, it is adviseable to empty the vessels of the part, by leeches or cupping, prior to its application; and I may be excused in hazarding an observation, from experience, that patients with dark skin, hair, and eyes, seem to be relieved much less frequently than those of a contrary temperament.

*Fructus fuci Vesiculosi usti.*—Take of the receptacles of the Fucus, before they are quite ripe, or are distended with the mucus, any quantity; wash in salt water; put them into a crucible on the fire, and occasionally stir with an iron rod until the mass becomes ignited to a dark red colour; then take, powder, and keep it in a well-closed glass vessel. This preparation produces a much more uniform one than that directed by Dr. Russel, and copied from him into most of our Dispensatories and Pharmacopœia. He gives it a high character, and calls it "Æthiops vegetabile," and used it successfully instead of burnt sponge, which it far exceeds in virtue. The dose will be from half a drachm to two drachms, two or three times a-day. It renders the bowels open and regular, promotes the appetite, and powerfully assists in removing glandular obstructions. It has been strongly praised in cases of hæmoptöe; but my experience does not warrant this character, excepting as to its laxative effect. I have not found any medicine so uniform in its effect, in cases of internal hæmorrhage, as the diluted muriatic acid, repeatedly given in the day; during which period, should the bowels become confined, I generally prefer this powder to correct them. As a vermifuge, it is less certain than the Pulvis fuci; and, in relieving acidity of the stomach, a few grains has been known to effect what the common medicines have failed to do. Externally applied to ulcers with a sanious discharge, it stimulates to healthy action; and, in a case of lepra extending over the extremities, I think large doses have been of service. As a powder for the teeth, the occasional use may be advantageous; but I have thought it injured the teeth and gums when constantly applied, probably from a portion of the alkali being in a state of subcarbonate.

### Fucus Nodosus.

*Fucus Nodosus:* frond coriaceous, compressed, veinless, subdichotomous; branched in a pinnated manner; receptacles distichous, pedunculated, roundish; mostly solitary; root a large shapeless, hard, woody, mass, inclining generally to conical or roundish.

Fronds numerous, from the same base; compressed, or between flat or compressed; destitute of midrib or veins; from two to four, or even six, feet long; about three lines wide, and every where linear, except that they are here and there swollen into vesicles, whence the plant derives a knotty appearance and name. They rise to the height of some inches undivided, and are then forked, after which they are generally twice or thrice dichotomous, with parting segments, and, besides these, furnished with lateral horizontal branches, more or less plentiful in different specimens, some crowded, others remote; some a foot or two long, others not as many inches; some simple, others forked. All the branches are remarkably attenuated at their bases, so as to be attached only by very slender points, and are, along their margin, serrated with extremely minute alternate distinct teeth, from the axilla of which would arise new branches, if the age of the plant permitted it. At their apices they are blunt, and often terminated by minute vesicles. The smaller ones look like linear leaves; and it not unfrequently happens that specimens are found with a simple stem, merely lined with these, the appearance of which is quite pinnated. The vesicles are innate in the frond, of which they occupy the whole substance, and are considerably wider than it in size. They vary from that of a hazel-nut to that of a walnut. They are always solitary, and mostly perforated by intervals of some inches. Externally they are smooth, internally hollow, and quite empty, except that their sides are lined by a coat of white pointed fibres.

Fructification spherical or elliptico-spherical; receptacles, some not larger than a pea, others as large as an acorn, scattered along the sides of the branches, and coming out of the axilla of the seratures; generally solitary, but sometimes two or three together, supported upon compressed peduncles, that gradually widen from very narrow bases, and half an inch or an inch long; the exterior of the receptacles is slightly uneven, and perforated all over with minute pores, under which lie imbedded spherical tubercles, composed of short simple pointed fibres, mixed with roundish brown seeds, which have a transparent limbus, and with pellucid elliptical granules, many times smaller than the seeds, similar to those observable in Fucus tuberculatus, and some other species of Fuci; the internal part of the receptacles is filled with a colourless mucus, through which passes a net work of white pointed fibres, with irregular masses; colour olive green, glossy when fresh, not in the least transparent, yellow in the receptacles, but every where black after it is dried; substance coriaceous, flexible, extremely extremely rough; a line, or a line and a half, thick, in the primary part of the frond.

The Fucus nodosus is a plant almost as common, and growing in nearly the same situations, on our shores, as the F. vesiculosus; and is occasionally to be met with in some places where the F. vesiculosus does not grow, at least not in such perfection as to fit it for medical purposes. The fruit of the F. nodosus provides us with a valuable substitute for that of the vesiculosus, and, in one respect, has an advantage, and vegetates and comes to perfection much earlier in the year, as it may be collected in January and February. This plant is more greedily sought for by the manufacturers of kelp, as its combustion produces a larger proportion of soda than any of the other marine plants. As a substitute for the Fucus vesiculosus, it may be prepared according to the directions given for the latter, attending, with equal care, to selecting the proper sorts.

*Worcester House, Swansea;*
*August* 1, 1816.

---

*For the London Medical and Physical Journal.*

